# The NSEBA Demonstration Project: implementation of a point-of-care platform for early infant diagnosis of HIV in rural Zambia

**DOI:** 10.1111/tmi.13627

**Published:** 2021-06-07

**Authors:** Catherine G. Sutcliffe, Nkumbula Moyo, Jessica L. Schue, Jane N. Mutanga, Mutinta Hamahuwa, Passwell Munachoonga, Sylvia Maunga, Philip E. Thuma, William J. Moss

**Affiliations:** 1 Johns Hopkins Bloomberg School of Public Health, Baltimore, MD, USA; 2 Macha Research Trust, Choma, Zambia; 3 Livingstone Central Hospital, Livingstone, Zambia

**Keywords:** early infant diagnosis, HIV, paediatrics, point-of-care testing, rural health services, sub-Saharan Africa

## Abstract

**OBJECTIVES:**

To describe the experience and resource requirements of implementing point-of-care testing for early infant diagnosis of HIV in rural Zambia.

**METHODS:**

A demonstration project was conducted using a hub-and-spoke model in 2018–2019 at five clinics in rural Zambia. Two testing hubs were established, and all HIV-exposed infants were tested with the GeneXpert system. Data on costs, turnaround times and test results were collected.

**RESULTS:**

Seven hundred and eighty six tests were conducted. At the hubs, results were available a median of 2.4 (IQR: 2.1, 2.8) hours after sample collection and most mothers (84%) received same-day results. At the spoke facilities, results were available a median of 9 days (IQR: 7, 12) after sample collection and provided to the mother a median of 16 days (IQR: 10, 28) after sample collection. Eleven children tested positive, and 9 (82%) started treatment a median of 13 days (IQR: 7, 21) after sample collection and on the day mothers received results. In contrast, results from matching samples sent for routine testing were available a median of 38 days (IQR: 27, 61) after sample collection and provided to the mother a median of 91 days (IQR: 47, 135) after sample collection.

**CONCLUSIONS:**

Implementing point-of-care testing in a network of rural health centres in Zambia required significant initial and ongoing investment in infrastructure, training and supervision. However, point-of-care testing can rapidly diagnose HIV-infected infants, so they can benefit from early treatment.

## Introduction

In 2019, an estimated 150 000 children were newly infected with HIV globally, including 126 000 in sub-Saharan Africa and 6000 in Zambia [1]. Early diagnosis of HIV infection is critical because without treatment up to half of infected children will die by 2 years of age [2]. However, only 69% of HIV-exposed infants in eastern and southern Africa and 33% of HIV-exposed infants in western and central Africa were estimated to have received a virologic test by 8 weeks of age [1]. With regard to treatment, only 58% of children living with HIV in eastern and southern Africa and 33% of children living with HIV in western and central Africa were receiving antiretroviral therapy (ART) [1].

Early infant diagnosis of HIV infection (EID) is challenging in sub-Saharan Africa as virologic tests must be performed, and these are primarily available in centralised laboratories in urban areas. The use of dried blood spots has increased access to virologic testing, but samples must be sent to central laboratories and results returned to the health facilities. This process provides many opportunities for delays, leading to poor retention in care and delays in diagnosis and treatment. This is particularly true in rural areas, where samples must be transported long distances to the laboratory and patients must travel long distances to the health facilities. In a recent multi-country evaluation of EID in sub-Saharan Africa (Cameroon, Cote d’Ivoire, Kenya, Lesotho, Mozambique, Rwanda, Swaziland and Zimbabwe), the median time from sample collection to return of results to the mother was 55 days [3]. Longer times of up to 125 days have been reported from both urban and rural areas [4–7]. In Zambia, delays in returning results to the facility after testing at birth led to as many as 48% of mothers failing to receive the results [5].

Point-of-care tests have been developed that can decentralise EID, thus reducing many of the steps and delays in the testing process [8]. Two tests, the m-PIMA (Abbott Laboratories, Forest Park, IL; formerly Alere Q) and GeneXpert (Cepheid Inc., Sunnyvale, CA), both based on nucleic acid detection, are commercially available and on WHO’s list of prequalified diagnostic tests [9]. Use of these tests was found to significantly reduce the turnaround time for results [3, 10], with more than 70% of mothers receiving results on the day of testing [4, 11–13]. In addition, point-of-care testing has been found to be cost-effective in several studies, particularly if multi-disease testing is performed [3, 14–16]. While these studies have documented the improved efficiency of point-of-care testing to rapidly return results and link infected infants to care, only a few studies report on practical experiences with implementing point-of-care testing, specifically the resources required to initiate and maintain a programme [12, 17]. This information is needed from diverse settings to guide programmes on the resource requirements when adopting these platforms.

A demonstration project was conducted in a small network of clinics in rural Zambia to (1) describe the personnel, infrastructure, and financial resources required to implement point-of-care (or near point-of-care) testing for EID; (2) evaluate the efficiency of point-of-care testing in terms of turnaround times; and (3) evaluate the feasibility of using the platform for multi-disease testing in this setting. The goal of the project was to provide evidence to stakeholders in Zambia and similar countries on the implementation of point-of-care testing for EID.

## Methods

### Overview and setting

The NSEBA Demonstration Project was conducted in Choma District, Southern Province, Zambia, in 2018–2019 and was nested within the Novel Screening for Exposed Babies (NSEBA) Study. In Southern Province, the HIV prevalence was estimated to be 13.3% among adults 15–59 years of age in 2016 [18]. At the time of the study, universal treatment of HIV-infected pregnant women was the standard of care [19], and testing for EID was recommended at birth, 6 weeks and 6 months of age with a nucleic acid test, and at 9, 12 and 18–24 months of age and at least 6 weeks after breastfeeding cessation with a serologic test [19]. In the study area, the standard of care was for DBS samples to be collected at the clinics and transported by the Ministry of Health to a central laboratory at Choma General Hospital for EID testing (COBAS^®^ AmpliPrep/COBAS^®^ TaqMan^®^ Systems [Roche Diagnostics, Risch-Rotkreuz, Switzerland]). Hardcopy results were transported back to the clinics using the same transport system. As the time for results to return to the clinics was variable, mothers typically received the result at their next clinic visit. In the event of a positive result, mothers were contacted by clinic staff to return as soon as possible.

### Study procedures

**Figure 1:**
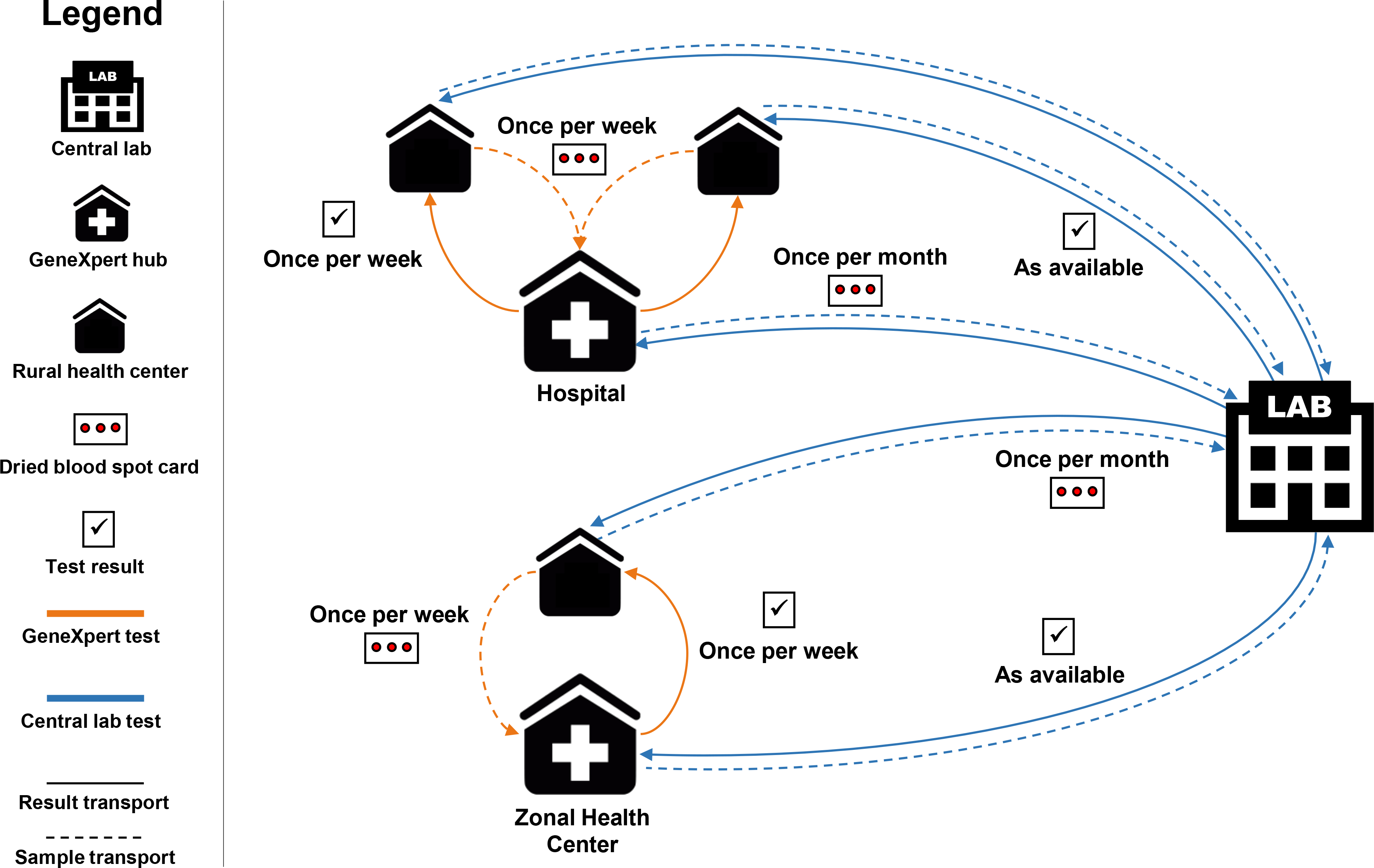
Study flow chart. Hub-and-spoke model for early infant diagnosis of HIV at five clinics in rural Zambia.

The NSEBA Study was previously described [5]. Briefly, the NSEBA Study was conducted to evaluate strategies for implementing point-of-care technologies for early infant diagnosis in Zambia. The study was conducted from February 2016 to March 2020 at clinics in the catchment area of Macha Hospital and the cities of Choma and Livingstone. The nested NSEBA Demonstration Project was conducted from 1 September 2018 to 31 December 2019 at only the five clinics in the catchment area of Macha Hospital (Figure S1). All HIV-exposed infants attending the study sites for early infant diagnosis and requiring a nucleic acid-based test were eligible to participate and were followed to their post-weaning visit or 31 December 2019. HIV-exposed infants were identified by study staff in the maternity wards or at the maternal and child health clinics, and their mothers were approached for participation. If willing to participate, mothers were asked to provide written informed consent and administered a questionnaire to collect information on demographics, antenatal care, HIV testing history and receipt of antiretroviral drugs. A blood sample was collected from the infant on a dried blood spot (DBS) card and sent for testing at the central laboratory as part of routine clinical care (Figure 1). At the same time, a second blood sample was collected for testing with a point-of-care (or near point-of-care) test.

### Point-of-care testing

The NSEBA Demonstration Project was conducted using the GeneXpert platform (Cepheid Inc, Sunnyvale, CA) (see Figure S2 for testing algorithms). This platform was selected as it was in use at health facilities around the country for diagnosis of tuberculosis and expansion to EID testing was being considered. A hub-and-spoke model was adopted for the five clinics included in this study (Figure 1). Two clinics with existing laboratories were identified as hubs: (1) A district level hospital that had a GeneXpert IV used for the diagnosis of tuberculosis and lab technicians trained in its use. However, the study was not permitted to use the machine for HIV testing; therefore, this evaluation was conducted in the research laboratory by study staff previously trained in its use. During the study period, the GeneXpert IV in the research laboratory was also used for HIV viral load testing for all paediatric patients in care at the HIV clinic. (2) A zonal health centre (a larger health centre with a laboratory that has the capacity to perform basic tests) with no previous experience with GeneXpert. A GeneXpert IV was purchased and placed in the clinic laboratory, and the lab technicians were trained by the Cepheid vendor from Lusaka. EID and HIV viral load testing were performed for all paediatric patients.

At the hub facilities, the GeneXpert test was used as a point-of-care test. A liquid whole blood sample was collected from the infant in an EDTA blood collection tube (Microvette^®^ K3 EDTA, Sarstedt AG & Co, Germany) and transported to the laboratory where it was processed and tested the same day. EID testing was prioritised, such that EID samples were tested immediately upon arrival at the laboratory. Results were delivered to the clinic to be given to the mother, with the goal of same-day delivery.

The three remaining rural health centres were designated as spoke facilities to each hub based on proximity and ease of sample transportation. At the spoke facilities, the GeneXpert functioned as a near point-of-care test. As sample transport was only feasible on a weekly basis, a DBS card was collected and stored at room temperature. The samples were transported once a week by the study team to the hub facility, and results from the previous week were returned. The goal was to provide results to the mother within two weeks of sample collection. Mothers were given appointments to return to the clinic (usually 2 weeks after sample collection) or they were contacted by phone or through community health workers when results were available and requested to return to the clinic to receive them.

Results from GeneXpert testing were documented as well as the dates and times for each step in the testing process and any resulting linkage to care and ART initiation. Consistent with the standard of care, when results from the central laboratory were returned, they were provided to the mother at their next clinic visit if concordant with the GeneXpert result. If the result from the central laboratory was discordant, the mother was contacted to return to the clinic. Results from the central laboratory were documented as well as the dates for each step in the testing process. At the end of the study period, an attempt was made to retrieve results that were not returned from the laboratory by examining the lab database.

All costs associated with implementing GeneXpert testing, including upgrading the laboratories and purchasing and maintaining equipment and supplies, were documented during the study period.

### Statistical analysis

Descriptive statistics, including proportions for categorical variables and medians, interquartile ranges and ranges for continuous variables, were used to summarise the data overall and by hub. Implementation data, including costs and supervisory visits, were summarised. The study population was described in terms of maternal and child characteristics. Testing data, including the number of tests, proportion of tests returned within the target time frame (same day for hub and within two weeks for spoke facilities), turnaround times from sample collection to return of results to the clinic and mother, and test results, were summarised. The proportion of mothers receiving the results on the same day at the hub facilities were compared by year and location of testing using a chi-square test. Turnaround times were compared between the hub and spoke facilities using a Wilcoxon rank-sum test. The analysis was performed using Stata Statistical Software, Version 16 (StataCorp LP, College Station, TX).

### Ethics statement

The NSEBA Study was approved by the Institutional Review Boards at Macha Research Trust, the Johns Hopkins Bloomberg School of Public Health and the National Health Research Authority in Zambia. The NSEBA Demonstration Project was conducted after consultation with the Ministry of Health of Zambia. All parents or guardians provided written informed consent for participation in the study. As GeneXpert is approved as a diagnostic test for HIV by the WHO, results were provided to healthcare providers and caregivers and used for clinical decision-making.

## Results

### Implementing GeneXpert testing

The hospital hub was already set up to perform the assay, so testing was initiated immediately and all spoke facilities were initially designated to send samples to the hospital hub. The health centre hub required upgrades to the laboratory before testing could begin, including installation of ceiling boards and an air conditioner, and upgrades to plumbing and electrical fittings and door closures (Table 1). In addition, the lab technician required training on how to use the machine and perform the assay (Table 1). Testing whole blood samples at the health centre hub began in November 2018. Additional equipment and training were required to perform the assay with DBS samples, which was not completed until February 2019. By this time, a second lab technician was working at the health centre hub who was also trained. In February 2019, the designated spoke facility began sending DBS samples to the health centre hub.

During the evaluation, repairs were required for both the laboratory and the GeneXpert equipment at the health centre hub (Table 1 and Table S1), including servicing of the air conditioner on three occasions, a computer malfunction and a printer malfunction. The computer and printer were both sent to the vendor in Lusaka for repair, and a back-up computer and printer were installed in the laboratory to continue testing. In addition, the GeneXpert machine at the hospital hub had to be taken to the vendor for repair.

As testing was new to the lab technicians at the health centre hub, a plan to provide support and supervision was created and carried out by the lab technicians at the hospital hub. This included weekly visits to the health centre laboratory in the first month of testing and then monthly visits thereafter or as needed. Twenty-four scheduled visits were conducted during the study period, and five issues were identified and resolved (see Table S1 for a list of visits and issues). Seven phone calls and unscheduled visits occurred, primarily related to issues with the GeneXpert software and hardware.

### Testing and turnaround times

**Figure 2:**
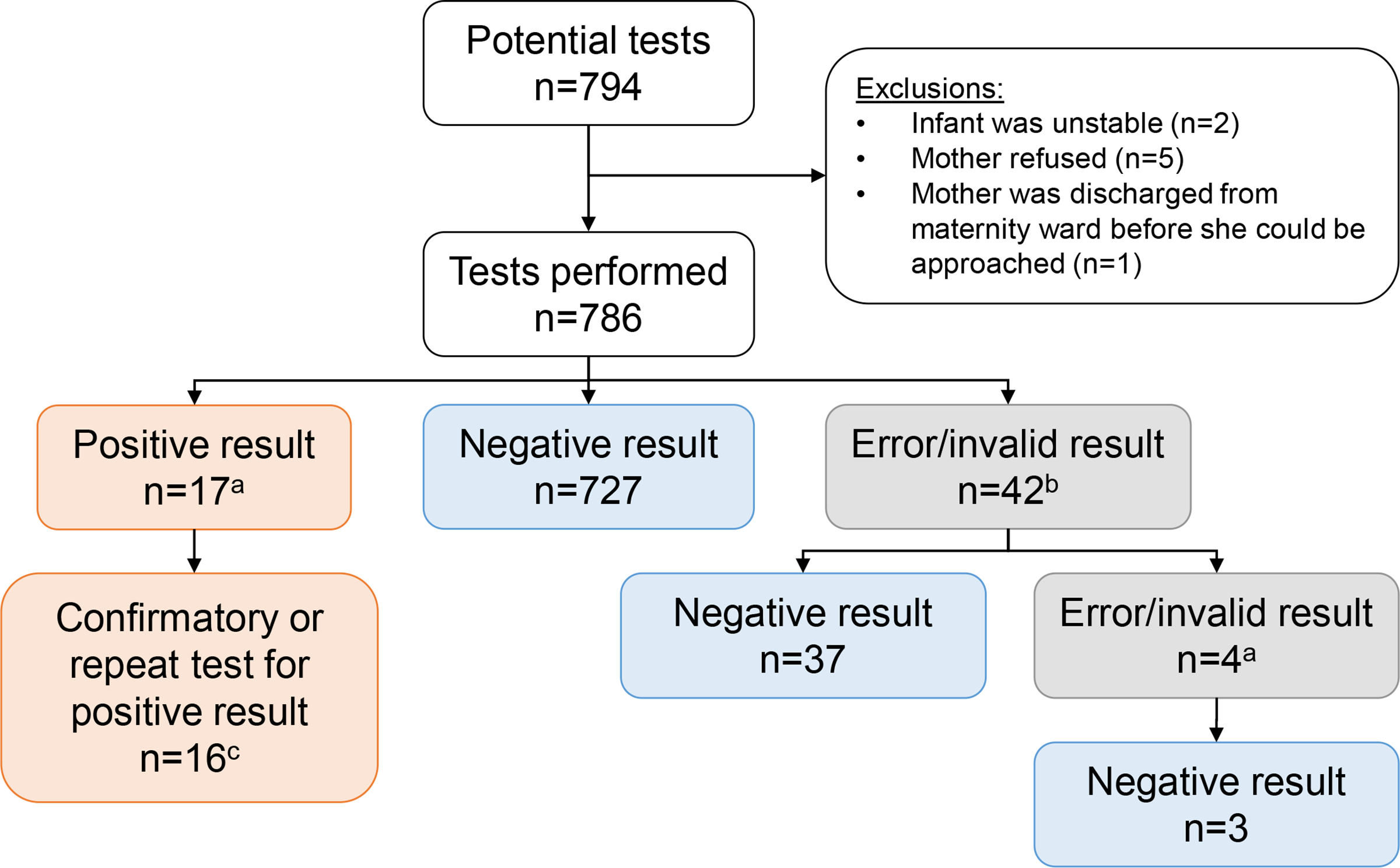
Summary of tests performed for early infant diagnosis in rural Zambia, 2018–2019. ^a^the 17 positive tests included either a confirmatory test with a new liquid whole blood sample on the same day or repeat testing with a new spot from the same dried blood spot card. ^b^1 sample had insufficient volume to perform another test. ^c^For 1 sample the mother refused a confirmatory test.

During the study period, 794 opportunities for testing were identified (Figure 2) and 786 tests (99.0%) were conducted on 421 children, half of whom were female (51.8%) and most of whom (92.8%) were born to mothers who received antiretroviral drugs for PMTCT (Table 2). The median age of the children tested was 1.61 months (IQR: 0.03, 6.13; range: 0, 23.1). Most tests were conducted at the hospital hub (75.0%), and just over half (56.6%) were conducted with liquid whole blood samples (Table 2). DBS samples were occasionally required at the hub facilities (2.7% and 21.4% of tests at the hospital and health centre hub, respectively) if lab technicians were not available for testing or there was a power outage such that testing could not occur on the same day as collection.

At the hubs, testing samples for EID was prioritised such that results were available at the clinic a median of 2.4 hours (interquartile range [IQR]: 2.1, 2.8) after sample collection (Table 3 and Figure S3). Most mothers (81%) waited at the clinic and received the results the same day. The proportion of mothers receiving results on the same day was higher in 2019 than 2018 (83% *vs.*71%; *P* = 0.02), and for children tested after birth in the maternal and child health clinics than at birth in the maternity ward (85% *vs.* 71%; *P* = 0.0004; Figure S4). At the spoke facilities, results were available at the clinic a median 9 days (IQR: 7, 12) after sample collection. Mothers received the results a median of 16 days (IQR: 10, 28) after sample collection, and only 43% of mothers received results within the two-week target window (Table 3 and Figure S3). In contrast, results from matching samples sent to the central laboratory for routine diagnosis were available at the clinic a median of 38 days (IQR: 27, 61) after sample collection and provided to the mother a median of 91 days (IQR: 47, 135) after sample collection.

A total of 830 cartridges were used to obtain a final result for the 786 tests (error rate = 44/786 = 5.6%; an additional 16 cartridges were used to confirm or repeat a positive result from the same day or sample). The final result was positive for 17 tests, negative for 767 tests and invalid for two tests (Figure 2). The 17 positive tests were for 11 children (Table 4), nine (81.8%) of whom started ART a median of 13 days after sample collection and the same day their mother received the results. Two mothers at the hub facilities refused care.

Results from the central laboratory were received and available for comparison for 729 tests (92.7%). Fifty-two samples (6.6%) were lost at the central laboratory, one sample was not sent as the child was found to have been recently tested, and four results (0.5%) were not available by completion of the study. Discrepant results were found for four tests (0.5%) from four children who were negative by GeneXpert and positive by the central laboratory. All four children were later determined to be uninfected on repeat testing (Table S2), consistent with laboratory errors at the central laboratory resulting in false positive test results. ART was initiated for two of the children after the positive result was received from the central laboratory but was later stopped after one and four months of ART.

During the study period, the GeneXpert machines at the hubs were also used for tuberculosis testing for all patients (hospital hub only) and HIV viral load testing (both hubs) for paediatric patients seen at those facilities. In addition to EID, on average the hospital hub performed 37 tests for tuberculosis and 38 HIV viral load tests per month, and the health centre hub performed 2 HIV viral load tests per month (Table 5). The lab technicians and machines were able to accommodate this level of testing and to prioritise EID testing to ensure that results were available on the same day for participants seen at the hub facilities.

## Discussion

This project demonstrated the public health importance of point-of-care platforms to improve the timeliness of EID in a rural area as well as the resources required to implement the programme using a hub-and-spoke model. In addition to investing in the point-of-care technology and assays, implementing the programme required that lab facilities be upgraded and maintained, and the health centre hub required ongoing support and supervision for the duration of the project. With this investment, implementing point-of-care testing improved EID testing, resulting in shorter turnaround times for results for all children and ART initiation for children testing positive.

Consistent with other studies [3, 4, 10, 11, 13], use of a point-of-care test dramatically decreased turnaround times for results and ART initiation at all facilities. In this rural area, the proportion of mothers receiving results on the same day at the hub facilities (81%) was lower than that reported from a trial in Mozambique (98.2%) and an observational study in Malawi (99.5%) [4, 11], and more similar to an evaluation of government programmes conducted in six African countries (72%) [13]. Willingness to wait for results was lowest in the first few months of the project when mothers were less familiar with point-of-care testing, and for testing at birth in the maternity wards when mothers desired to return home. However, even in the later months and for testing at other ages, only 80–85% of mothers received results on the day of testing. In addition, the median turnaround time for the spoke facilities (9 days) was longer than that reported from two multi-country evaluations using a hub-and-spoke model (2–7 days) [3, 10] due to the transportation schedule established for this project that took into account clinic volumes and logistics. Implementation of a text-based platform for returning results to the clinics could shorten these times for the spoke facilities [20, 21]. Overall, these results indicate that rural programmes may not see the full benefit of these assays due to challenges accessing care in these settings. However, these same challenges exacerbate the delays in diagnosis associated with centralised testing, and so rural programmes can still expect a significant improvement in the EID testing process with point-of-care or near point-of-care testing.

The decrease in turnaround times for results led to rapid ART initiation for children testing positive, the ultimate goal of point-of-care testing given the clinical and survival benefits of early treatment [22]. Prior to this project, median times to ART initiation in this area were 43 to 103 days with centralised testing and 4–11% of HIV-exposed children died before results were returned [6, 21]. With point-of-care testing, this interval was reduced to one day at the hub facilities and 15 days at the spoke facilities. Similar decreases have been observed in other studies [3, 4, 10, 11, 13].

This study highlighted the challenges of centralised testing, not only in lengthy turnaround times for results to return to the clinic, but also for results to get to the mother and to be used for clinical decisions. With point-of-care testing, almost all mothers received their child’s test results during the study period. In comparison, 6% of samples were lost on their way to or at the central laboratory and thus would not have generated results, and prior studies in this area suggest that 13–30% of mothers did not receive the results from testing at the central laboratory [5, 23]. In addition, this study identified four children who were misdiagnosed based on testing by the central laboratory, representing only 0.5% of all tests but 27% of children with a positive result by either test. False positives are to be expected given that nucleic acid-based testing is not 100% specific, and thus, confirmatory testing is both cost-effective and recommended [24]. However, the long turnaround time for results led to unnecessary exposure to ART that could have been avoided with point-of-care testing.

Implementing point-of-care testing and achieving the clinical and survival benefits required significant investment in personnel, infrastructure and financial resources. Use of the GeneXpert platform in this project necessitated that the new health centre hub facility be upgraded and maintained to satisfy the requirements for testing, as has been seen in other studies [17, 25]. While these facility upgrades may not be necessary with other available or future point-of-care tests, the ongoing maintenance of the platforms, initial and ongoing training needs, and ongoing supervision associated with testing will be relevant for all platforms and will need to be factored into implementation plans. The new health centre hub benefited from the expertise and experience of the hospital lab technicians, particularly in the first few months of the project. Given the low volume of tests performed each week, it took time for the new lab technicians to become comfortable with the assay despite the initial training, and it was helpful to have experts nearby who could provide support. Programmes implementing point-of-care testing should consider incorporating supervision into their plans, which could be provided by a network of designated ‘expert’ hubs. Another activity that this study did not implement but may be needed is an external quality assurance programme to ensure the accuracy of testing. Investing in such a programme was found to be cost-saving for several countries in sub-Saharan Africa when considering the costs of missed infections and unneeded treatment for false diagnoses [26].

Several studies conducted in sub-Saharan Africa have found point-of-care testing to be cost-effective in comparison with centralised testing, in terms of cost per test result returned within 30 days [3], years of life saved [14], additional children initiating ART within 60 days [15, 16] and deaths averted [15, 16]. To increase cost-effectiveness and achieve the full benefits of existing point-of-care platforms, programmes should implement multi-disease testing [15, 16]. Many countries, including Zambia, have GeneXpert machines at many health facilities for the diagnosis of tuberculosis and use could be expanded to EID and treatment monitoring for HIV-infected persons. This would, however, require collaboration between programmes at the administrative level and for facilities to devise plans for performing the different tests. As few studies have been conducted to inform implementation of multi-disease testing [17], this demonstration project was originally designed to so. However, we were unable to obtain permission to use the existing machines to conduct testing for both tuberculosis and HIV and testing for HIV viral load was limited to only paediatric patients. The results demonstrate that multi-disease testing was feasible with the volume of tests needed in this rural setting and that results for EID could be rapidly returned to the clinic and caregiver if EID testing was prioritised, as found in other studies [13, 17].

Several additional limitations to this demonstration project should be noted. First, a relatively small number of tests were conducted and only a small number of HIV-infected children were identified. This is a challenge for all studies of EID given the widespread availability of ART and high coverage of PMTCT in this region that has resulted in a dramatic decline in mother-to-child transmission [27]. Second, this project was conducted in a limited number of facilities in one rural area of Zambia. While the specific estimates may not be generalisable to all areas, the overall results and challenges identified are consistent with other studies and can be used to inform programme implementation plans. Lastly, this project was carried out with dedicated staff and funds and so may overestimate the impact of point-of-care testing. For example, study staff were tasked with managing stocks of testing kits and a back-up GeneXpert machine was available which allowed testing to continue throughout the project period without stockouts of reagents or periods of machine failure, challenges identified in other studies [12]. The results of this demonstration project can be viewed as the maximum benefits achievable with the resources invested in the programme.

## Conclusions

In summary, implementing point-of-care testing in a network of rural health centres in Zambia required significant initial and ongoing investment in infrastructure, training and supervision. However, point-of-care testing can rapidly diagnose HIV-infected infants so they can receive the benefits of early care and treatment.

## Supplementary Material

tS1-S2 **Table S1.** Supervisory visits to new hub at the Zonal Health Centre.
**Table S2.** Testing and treatment histories for children with discrepant results between GeneXpert and the central laboratory.

fS1-S4 **Figure S1.** Map of study area.
**Figure S2.** Algorithms for testing with GeneXpert with (A) liquid whole blood and (B) dried blood spots.
**Figure S3.** Time from sample collection to return ofresults to mothers for (A) all samples; (B) samples tested at the hospital hub; and C) samples tested at the health center hub.
**Figure S4.** Proportion of mothers receiving results on the same day at the hub facilities by (A) year and month; and (B) location of testing.

## Figures and Tables

**Table 1:** Costs associated with establishing and maintaining point-of-care testing for early infant diagnosis at one health centre in rural Zambia, 2018–2019

	Estimated cost in USD ^†^
Infrastructure costs	
Upgrades to the laboratory before study start	
Plumbing and electrical fittings	311
Air conditioner	915
Ceiling board and welding	301
Door closure and rubber seals	158
Labour charges	144
Transport and logistics	171
Other maintenance and repairs during study	
Electrical repairs	18
Air conditioner service/repair	25
Subtotal infrastructure costs	2043
Point-of-care testing costs	
GeneXpert equipment	
GeneXpert IV	17 000
Printer	250
UPS	790
Xpert check kit	450
Handling charges (freight, customs, bank fees)	1350
Thermomixer‡	1244
Training costs	
Initial installation and training	1250
Initial training – additional costs for travel (Lab tech #1 in October 2018)	74
Second training session for Lab tech #2 (January 2019)	477
GeneXpert supplies and recurring costs	
Xpert annual service	1062
Xpert check kit	346
Labour charges, including travel and accommodation	538
Handling charges (freight, customs, bank fees)	177
EID Xpert cartridges (230 at 14.90 USD each)	3427
VL Xpert cartridges (30 at 14.90 USD each)	447
Handling charges (freight, customs, bank fees) for per cartridge orders	598
EDTA specimen containers (230 at 0.37 each)	85
Printer cartridge refill	87
Subtotal point-of-care testing costs	29 652
Total costs of the programme	31 695

† For items purchased in Zambia using ZMW, the exchange rate from June 2019 (mid-way through the study) of 13 ZMW = 1 USD was used.

‡ Required for performing the test assay with dried blood spots.

**Table 2:** Characteristics of children and samples undergoing point-of-care testing in rural Zambia, 2018–2019

	Hospital Hub	Health Centre Hub	Total
Tests			
Total *N*	590	196	786
Sample type, *n* (%)			
DBS	228 (38.6)	113 (57.6)	341 (43.4)
Whole blood	362 (61.4)	83 (42.4)	445 (56.6)
Age, *n* (%)^†^			
Birth	198 (33.6)	42 (21.4)	240 (30.5)
Postnatal	18 (3.0)	10 (5.1)	28 (3.6)
6 weeks	149 (25.2)	55 (28.1)	204 (26.0)
6 months	125 (21.2)	53 (27.0)	178 (22.6)
>7 months	100 (17.0)	36 (18.4)	136 (17.3)
Children			
Total N	325	96	421
Number of times tested, median (IQR; range)	1 (1, 2; 1–5)	2 (1, 3; 1–4)	2 (1, 2; 1–5)
Sex – female, *n* (%)	172 (52.9)	46 (47.9)	218 (51.8)
Mother received antiretroviral drugs for PMTCT, *n* (%)	303 (93.2)	88 (91.7)	391 (92.8)
Travel time (hours) to the clinic, median (IQR; range)	1.0 (0.8, 2.0; 0–22.0)	0.8 (0.5, 1.0; 0–4.0)	1.0 (0.7, 2.0; 0–22.0)
Mobile phone available, *n* (%)^‡^	121 (59.6)	57 (72.2)	178 (63.1)

DBS, dried blood spot; IQR, interquartile range; PMTCT, prevention of mother-to-child transmission.

† Birth = 0–6 days; postnatal = 7–28 days; 6 weeks = 4–13 weeks; 6 months = 3.0–7.0 months; >7 months = 7.1–23.9 months.

‡ Only available for children tested at birth (*n* = 188 at Hospital hub; *n* = 40 at zonal health centre hub).

**Table 3:** Turnaround times and return of results for point-of-care testing in rural Zambia, 2018–2019

Turnaround time from sample collection	Hospital Hub				Zonal Health Centre Hub				Total			
	Whole Blood (hours)		DBS (days)		Whole Blood (hours)		DBS (days)		Whole Blood (hours)		DBS (days)	
	*N*	Median (IQR; range)	*N*	Median (IQR; range)	*N*	Median (IQR; range)	*N*	Median (IQR; range)	*N*	Median (IQR; range)	*N*	Median (IQR; range)

To result available at clinic ^†^	356	2.4 (2.2, 2.8; 1.7, 75)	227	9 (7, 13; 0, 69)	83	2.2 (2.0, 2.9; 1.7, 26)	112	8 (7, 11; 0, 17)	439	2.4 (2.1, 2.8 1.7, 75)	339	9 (7, 12; 0, 69)
To result given to mother ^‡^	343	2.6 (2.3, 3.3; 1.7, 2830)	219	15 (10, 24; 1, 243)	83	2.4 (2.0, 4.2; 1.7, 938)	111	20 (11, 45; 1, 149)	426	2.6 (2.3, 3.4; 1.7, 2830)	330	16 (10, 28; 1, 243)

Result given to mother	*N*	*n* (%)	*N*	*n* (%)	*N*	*n* (%)	*N*	*n* (%)	*N*	*n* (%)	*N*	*n* (%)

On schedule ^§^	362	294 (81)	228	104 (46)	83	65 (78)	113	42 (37)	445	359 (81)	341	146 (43)
Within 30 days	362	334 (92)	228	184 (81)	83	81(98)	113	69 (61)	445	415 (93)	341	253 (74)
Ever	362	343 (95)	228	219 (96)	83	83 (100)	113	111 (98)	445	426 (96)	341	330 (97)

DBS, dried blood spot; IQR, interquartile range.

† *P* = 0.002 for whole blood and *P* = 0.005 for dried blood spot cards (DBS) for comparison between the hospital and zonal health centre hubs by Wilcoxon rank-sum test.

‡ *P* = 0.03 for whole blood and *P* = 0.007 for dried blood spot cards (DBS) for comparison between the hospital and zonal health centre hubs by Wilcoxon rank-sum test.

§ On schedule defined as on the same day as sample collection for whole blood and ≤14 days for DBS.

**Table 4:** Treatment initiation among children testing positive rural Zambia, 2018–2019

	Hub facilities	Spoke facilities	Total
Number of children testing positive	4	7	11
Number of children initiating ART	2	7	9
Time (days) from sample collection to ART initiation, median (IQR, range)	1 (1, 1; 1–1)	15 (11, 23; 7–55)	13 (7, 21; 1–55)
Time from mother receiving results to ART initiation	1 (1, 1; 1–1)	0 (0, 0; 0–25)	0 (0, 1; 0–25)

ART, antiretroviral therapy; IQR, interquartile range.

**Table 5:** Number and type of tests performed using the GeneXpert platform in rural Zambia, 2018–2019

	Hospital Hub				Zonal Health Centre Hub		
	TB^†^	EID	VL^‡^	Total	EID	VL^‡^	Total
September 2018	61	6	-	67	-	-	-
October 2018	70	27	-	97	-	-	-
November 2018	60	36	-	96	2	-	2
December 2018	30	40	-	70	2	-	2
January 2019	42	49	28	119	2	0	2
February 2019	58	39	48	145	17	1	18
March 2019	0^§^	42	35	77	17	0	17
April 2019	0^§^	32	67	99	18	2	20
May 2019	51	45	26	122	25	6	31
June 2019	38	24	19	81	15	1	16
July 2019	37	41	14	92	11	0	11
August 2019	37	50	34	121	18	1	19
September 2019	28	39	22	89	15	3	18
October 2019	25	47	17	89	19	4	23
November 2019	31	39	9	79	14	0	14
December 2019	37	34	3	74	21	6	27
Monthly average	38	37	37	95	14	2	14
Total	605	590	224	1517	196	24	220

EID, early infant diagnosis; TB, tuberculosis; VL, HIV viral load.

† TB testing was performed by hospital lab technicians using a Genexpert machine located at the Macha Hospital laboratory, while EID and HIV viral load testing were performed by research laboratory technicians using a GeneXpert machine located in the Macha Research Trust laboratory.

‡ HIV viral load testing for paediatric patients only was started on 21 January 2019.

§ The hospital laboratory ran out of GeneXpert cartridges and testing was paused.
