# Supplementary material for: The NSEBA Demonstration Project: implementation of a point-of-care platform for early infant diagnosis of HIV in rural Zambia
Source: Trop Med Int Health. Author manuscript; Available in PMC 2022 Sep 1. (PMC8416694; doi:10.1111/tmi.13627)
Supplement: tS1-S2 — Table S1. Supervisory visits to new hub at the Zonal Health Centre. Table S2. Testing and treatment histories for children with discrepant results between GeneXpert and the central laboratory. [file NIHMS1705244-supplement-tS1-S2.docx]

**Supplemental Table 1. Supervisory visits to new hub at the Zonal Health Center**

| **Type of supervision** | **Number** | **Comment** |
| --- | --- | --- |
| Scheduled visits | 24 | Issues identified at 5 visits:   1. Protocol deviation in conducting the test (second cartridge was loaded before the first finished running) 2. Computer malfunction – resolved by sending to the vendor 3. Loose module door – resolved with repair during annual maintenance visit by vendor 4. Air conditioner in need of repair at two visits – resolved with maintenance visit to repair the air conditioner |
| Phone calls and unscheduled visits | 7 | 1. Troubleshooting at first test – resolved with visit to the hub 2. Troubleshooting software – resolved with visit to the hub 3. Troubleshooting software – resolved over the phone 4. Printer cartridge low – new cartridge purchased 5. Printer not working – resolved by sending printer to the vendor 6. Air conditioner not working – resolved with maintenance visit to repair the air conditioner 7. Protocol deviation detected in sample processing – resolved with training over the phone |

**Supplemental Table 2. Testing and treatment histories for children with discrepant results between GeneXpert and the central lab**

|  | **Child 1** | **Child 2** | **Child 3** | **Child 4** |
| --- | --- | --- | --- | --- |
| **Sex** | Male | Female | Female | Male |
| **Mother receiving ART** | Yes | Yes | Yes | Yes |
| **Child received PMTCT** | Yes | Yes | Yes | Yes |
| **First test** |  |  |  |  |
| Age | 7 days | 11.6 weeks | 1 day | 6 days |
| GeneXpert result | n/a | Negative | Negative | Negative |
| SoC result | Negative | Positive | Negative | Sample lost |
| **Second test** |  |  |  |  |
| Age | 7.0 weeks | 5.9 months | 8.9 weeks | 5.9 weeks |
| GeneXpert result | n/a | Negative | Negative | Negative |
| SoC result | Negative | Negative | Positive | Negative |
| **Third test** |  |  |  |  |
| Age | 5.9 months | 8.6 months | 3.9 months | 6.0 months |
| GeneXpert result | Negative | Negative | n/a | Negative |
| SoC result | Positive | Negative | Negative | Positive |
| **Fourth test** |  |  |  |  |
| Age | 7.8 months | n/a | n/a | 8.5 months |
| GeneXpert result | Negative | n/a | n/a | Negative |
| SoC result | Negative | n/a | n/a | Negative |
| **ART initiated** | Yes, after initial positive test | No, initial positive result misplaced and found after next negative test result was returned | Yes, after initial positive test | No, waited for results of confirmatory testing |
| **ART stopped** | Yes, after confirmatory test was negative. Child received one month of ART. | n/a | Yes, after consulting with district health office. Child received four months of ART. | n/a |

ART: antiretroviral therapy; n/a: not applicable; PMTCT: prevention of mother to child transmission; SoC: standard of care PCR testing at the central lab
