# Supplementary material for: The NSEBA Demonstration Project: implementation of a point-of-care platform for early infant diagnosis of HIV in rural Zambia
Source: Trop Med Int Health. Author manuscript; Available in PMC 2022 Sep 1. (PMC8416694; doi:10.1111/tmi.13627)
Supplement: fS1-S4 — Figure S1. Map of study area. Figure S2. Algorithms for testing with GeneXpert with (A) liquid whole blood and (B) dried blood spots. Figure S3. Time from sample collection to return ofresults to mothers for (A) all samples; (B) samples tested at the hospital hub; and C) samples tested at the health center hub. Figure S4. Proportion of mothers receiving results on the same day at the hub facilities by (A) year and month; and (B) location of testing. [file NIHMS1705244-supplement-fS1-S4.docx]

**Supplemental Figure 1. Map of study area**

RHC: rural health center

Map of Southern Province, Zambia. *Google Maps,* September 2019 (https://www.google.com/maps/place/Southern+Province,+Zambia/@-16.68371,25.8207546,8z/data=!3m1!4b1!4m5!3m4!1s0x1946958bdd263cdd:0xbff302af89f265b4!8m2!3d-16.9620634!4d26.419389)

**Supplemental Figure 2. Algorithms for testing with GeneXpert with A) liquid whole blood and B) dried blood spots**

**B**

**A**

**Supplementary Figure 3. Time from sample collection to return of results to mothers for A) all samples; B) samples tested at the hospital hub; and C) samples tested at the health center hub**

**Supplemental Figure 4. Proportion of mothers receiving results on the same day at the hub facilities by A) year and month; and B) location of testing**

Note: Comparison by year: p=0.02; comparison by location of testing: p=0.004
